# Covalent Adaptable Network and Self-Healing Materials: Current Trends and Future Prospects in Sustainability

**DOI:** 10.3390/polym12092027

**Published:** 2020-09-05

**Authors:** Ajmir Khan, Naveed Ahmed, Muhammad Rabnawaz

**Affiliations:** School of Packaging, Michigan State University, 448 Wilson Road, East Lansing, MI 48824-1223, USA; khanajmi@msu.edu (A.K.); ahmednav@msu.edu (N.A.)

**Keywords:** covalent adaptable networks, self-healing, recycling, future prospects, sustainability

## Abstract

This work estimates that if the growth of polymer production continues at its current rate of 5% each year, the current annual production of 395 million tons of plastic will exceed 1000 million tons by 2039. Only 9% of the plastics that are currently produced are recycled while most of these materials end up in landfills or leak into oceans, thus creating severe environmental challenges. Covalent adaptable networks (CANs) materials can play a significant role in reducing the burden posed by plastics materials on the environment because CANs are reusable and recyclable. This review is focused on recent research related to CANs of polycarbonates, polyesters, polyamides, polyurethanes, and polyurea. In particular, trends in self-healing CANs systems, the market value of these materials, as well as mechanistic insights regarding polycarbonates, polyesters, polyamides, polyurethanes, and polyurea are highlighted in this review. Finally, the challenges and outlook for CANs are described herein.

## 1. Introduction

**Figure 1:**
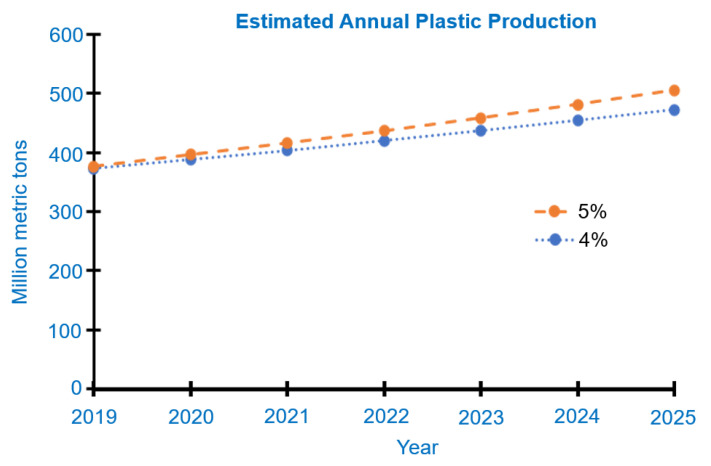
Estimated plastic production between 2019 and 2025. The annual growth is projected both at 4% and 5%.

Polymers are used for a myriad of applications. In 2018, the annual production of plastics reached 359 million tons [1]. The yearly growth of plastics is expected to be in the range of 4–5% [2,3,4]. We estimated the annual plastic production from 2019 to 2025, both at 4% and 5% yearly growth rate (Figure 1**)**. Approximately ~505 million tons of plastics will be produced in 2025. Based on 5% annual growth rate, plastic production will exceed 1000 million tons/year in 2039. Most of the polymers that are produced eventually end up in landfills and some of these materials contaminate the environment, thus posing daunting environmental, societal, and economic challenges. Thermosets, which account for more than 20% of the polymers used annually, end up in landfills as thermosets are difficult to recycle due to their permanent crosslinked structures. This review article is focused on the emerging crosslinked materials that behave as thermosets at room temperature but can be reused/recycled at the end of their lifecycle.

Chemical bonds (primary and/or secondary) that reversibly break and reform can be categorized into dynamic non-covalent interactions and dynamic covalent bonds. The dynamic non-covalent interactions are characterized by the rapid exchange of the secondary bonds in their networks. While this rapid exchange offers faster self-healing properties, but they often have poor mechanical properties and have low creep resistance [5]. Dynamic covalent bonds (DCBs) are reversible covalent bonds at elevated temperature but are stable at ambient temperatures [6]. DCBs network materials are getting more popular because of their dimensional stability, excellent self-healing properties, and their potential as sustainable alternatives for conventional thermosets as well as access to shape memory materials [7,8].

DCBs, when incorporated into polymer network materials, are referred to as covalent adaptable networks (CANs) [9]. These CANs bearing reversible bonds in their networks are categorized into two different types. Dissociative CANs, where a complete rupture of the existing bonds occurs upon heating and reformation of the bonds upon cooling. Diels-Alder chemistry is a well-known example of a dissociative CANs [10]. Associative CANs are those where existing covalent bonds are only broken when new ones are formed. Vitrimers is a special category of associative CANs, for which the criteria include: (i) they are covalently bound network of chains; (ii) their network topology can be changed by the exchange reactions, resulting in the thermal malleability of the network. These exchange reactions of CANs make them suitable for reprocessable thermosets [11,12,13].

**Figure 2:**
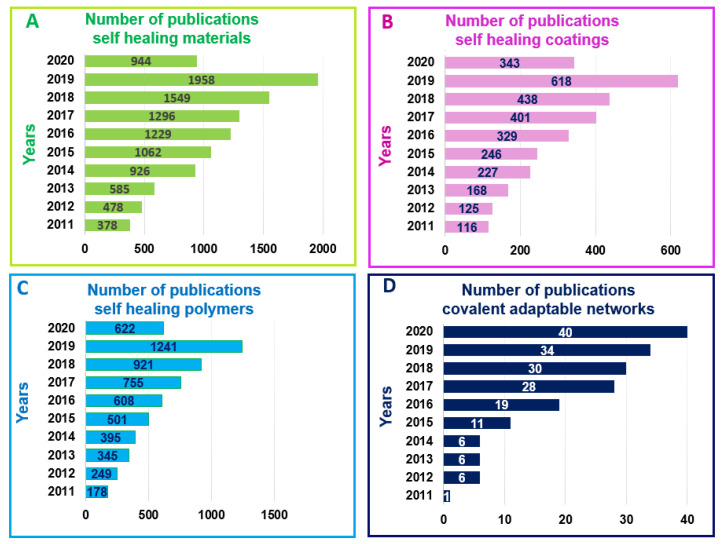
Articles published in the last 10 years related to: (**A**) self-healing materials; (**B**) self-healing coatings; (**C**) self-healing polymers and (**D**) covalent adaptable networks. These numbers were generated from the “Web of Science database” using the keywords “self-healing materials” for (**A**), “self-healing coatings” for (**B**)**,** “self-healing polymers” for (**C**), and “covalent adaptable networks” for (**D**). (Date data acquired: 15 July 2020).

The science of self-healing CANs is expanding rapidly due to the growing demand for sustainable materials [14]. Due to their promising potential applications, the number of research articles related to these materials is rapidly increasing (Figure 2). The self-healing theme is more common in literature than the CAN because the former covers covalent as well as non-covalent networks, while CAN covers only covalent networks. In addition, in the last three decades, the research focus has been mainly self-healing materials for coatings applications (e.g., anti-rust sector) where both intrinsic and extrinsic self-healing systems have been widely explored. In contrast, CAN is a newly emerging area. For example, only 6 publications for CANs were reported in 2014, and this number was 6-fold higher in 2019 (Figure 2D). On the other hand, the number of publications on self-healing systems increased by ~3-fold in the same period (Figure 2A–C). More direct evidence for the rapid growth of CAN compared to the self-healing materials is the number of articles published in 2020. In the case of CAN, the number of articles has already surpassed those of 2019, while self-healing is somewhere 50–60% of those reported in 2019. We anticipate excellent growth rate CANs articles in the future due to their applications in the numerous fields, ranging from recycling to flexible electronics and from soft robotics to wearable devices.

**Scheme 1:**
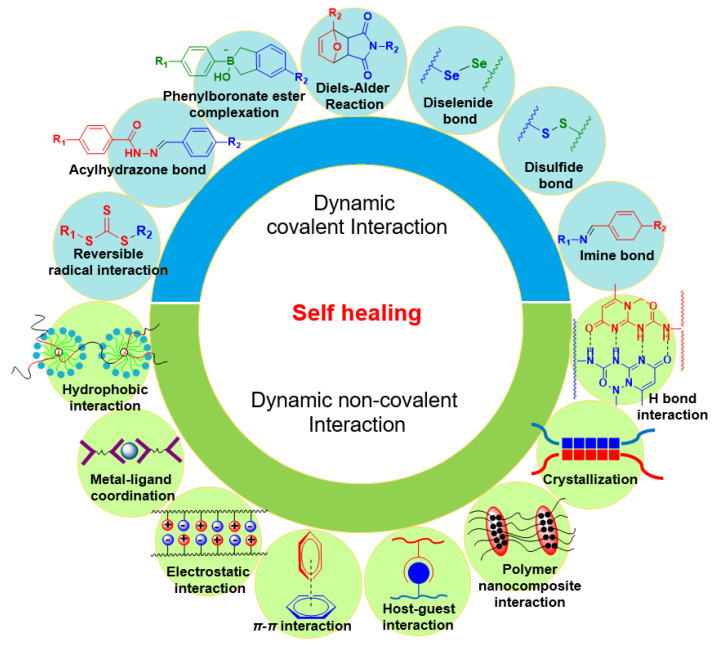
Examples of functional groups that are commonly used in covalent adaptable networks (CANs) and non-covalent adaptable networks (non-CANs) polymers.

The range of functional groups used to acquire reversibility is expanding rapidly, as can be seen in Scheme 1. The most commonly reported CANs utilized for the self-healing properties include Diels-Alder reactions [15,16], nitroxides [17], acylhydrazone bonds [18], disulfide-bonds [19,20], hemiaminal linkages [21], as well as those involved in ring-opening [22], and trans-esterification reactions [23]. Non-covalent adaptable networks (Non-CANs) such as those based on van der Waals forces [24], transition metal–ligand interactions [25], *π−π* interactions [26], host–guest chemistry [27], and hydrogen bonding [28,29], are also commonly reported to exhibit self-healing performance. Among the CANs reported, polyesters, polyamides, polycarbonates, polyurethanes, and polyureas are of great interest due to their widespread use. Therefore, this review article is focused on the use of CANs in the above systems with an emphasis on their growth trends, market analysis, mechanistic insights, potential applications, and outlook.

**Scheme 2:**
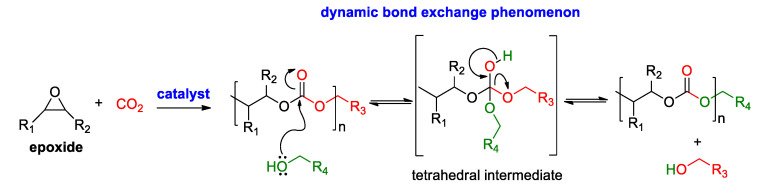
The synthetic route toward polycarbonates via ring-opening polymerization (ROP), and depiction of the dynamic bond exchange phenomenon. Reproduced from ref. [47] with permission from the American Chemical Society.

**Figure 3:**
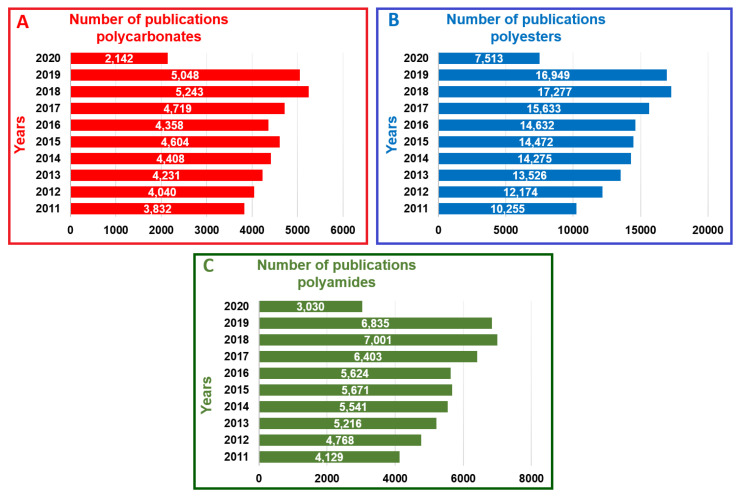
Articles published in the last 10 years related to: (**A**) polycarbonates; (**B**) polyesters; and (**C**) polyamides. These numbers were generated from the “Web of Science database” using the keywords “polycarbonates” for (**A**), “polyesters” for (**B**), and “polyamides” for (**C**). (Date data acquired: 15 July 2020).

**Transcarbonation**: Polycarbonates (PCs) are versatile materials that can easily be molded into various forms and shapes. Due to their unique mechanical properties and excellent thermal behavior, PCs are widely used in transportation, construction, packaging, electrical, and electronics optical media, medical devices, and airplane windows [30]. Due to widespread applications, the number of research articles related to PCs is increasing rapidly (Figure 3A). For example, ~46,235 articles have covered the topic of polycarbonates since 2010 to the mid of 2020. Approximately 6 million tons of PCs are produced annually [31]. According to a recent study, the global production of PCs is estimated to reach 7.72 million tons, with a value of USD 25.37 billion by 2024 [32,33]. Polyvinyl chloride (PVC) is getting controversial due to safety concerns. In addition, the recycling of PVC is challenging as it tends to form HCl as it degrades, which corrodes equipment during recycling processes. PCs are considered to be a natural replacement for PVC, and thus PCs are expected to become utilized in some markets that have traditionally relied on PVC. Traditionally, PCs are synthesized via the reaction of bisalcohols or biphenols with phosgene chloride [34]. PCs can also be synthesized via the copolymerization of epoxides with CO_2_ as an inexpensive, nontoxic, abundant, and renewable feedstocks [34,35] Scheme 2 depicts the synthesis of PCs from CO_2_ and epoxide via ring-opening polymerization (ROP) along with their CAN chemistry. Reversible covalent bonds in a polymer framework lead to self-healing, remoldability, plasticity, and potential recyclability. Some articles have been reported related to the dynamic bond in PCs [36,37,38,39,40,41,42,43,44,45,46]. In a similar matter, as occurs during transesterification, carbonates undergo transcarbonation exchange reactions with free hydroxyl groups [34,47]. The presence of free-hydroxyl groups is key for achieving dynamic exchange at the carbonate linkage.

**Scheme 3:**
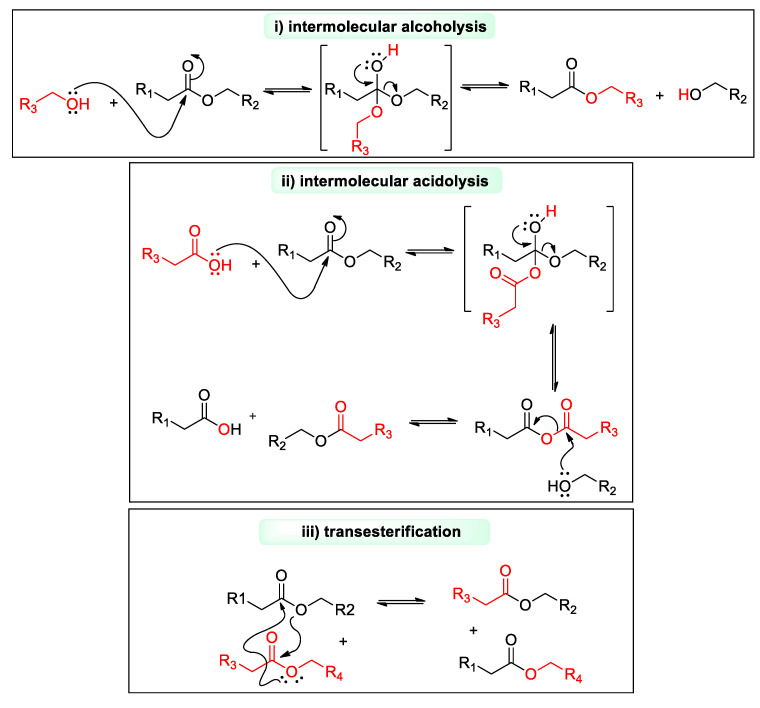
Mechanism of transesterification reactions involves in polyesters. This scheme is reproduced from ref. [58] with the permission of *Wiley* Publishers.

**Transesterification:** Polyesters are widely used as commodity and engineering polymers. In particular, they account for 8.5% of the market of all plastic materials, which holds the 5th place, while highest amount polymer used in the packaging industry in the US [1,48,49]. Overall, polyesters are known for their excellent mechanical stability and high resistance to shrinkage. Thanks to these properties, polyesters are used in the packaging and textile sectors as well as in many other industries. In 2018, polyester fiber production was estimated to be approximately 55.1 million metric tons [50]. Polyesters are sometimes crosslinked in order to improve their mechanical properties but unfortunately, this limits their recyclability. The presence of CANs as crosslinkers can help to facilitate the recycling of lightly crosslinked polyesters. The chemistry of transesterification reactions involving polyesters has been reported by various researchers [16,51,52,53,54,55,56]. For example, Kotliar has reviewed three basic mechanisms through which the dynamic bonds of polyesters interchange [57,58,59]. These three basic mechanisms are classified as: (i) intermolecular alcoholysis, (ii) intermolecular acidolysis, and (iii) transesterification, as illustrated in Scheme 3.

**Transamidation:** Transamidation is the exchange of amide bonds in polyamides for self-healing and recycling [58,60]. Repair and recycling of crosslinked polyamides has a multitude of benefits from both environmental and economic standpoints. Polyamides have excellent mechanical properties, including high impact strength (toughness), high tensile strength, good resilience, high flexibility, and low creep. Due to this versatility, polyamides are often used in textiles, carpets, sportswear, kitchen utensils, and in the automotive industry. Consequently, the transportation and garments manufacturing industries are the major consumers of polyamides. According to “GLOBE NEWSWIRE”, by 2026, the global market for the polyamide market is anticipated to be USD 38.3 billion [61,62]. Polyamides market is rapidly growing in various sectors; while the primary growth is witnessed in the automotive industry. However, polyamide production creates nitrous oxide (N_2_O), which is 300 times more potent than CO_2_ in terms of greenhouse effects [63]. Similarly, during the manufacturing of nylon, a large amount of water is used to cool the fibers, which can lead to pollution as well as placing a strain on global water supplies. Therefore, the recycling and reuse of polyamides will eliminate these environmental issues that are currently encountered with the production of polyamides.

**Scheme 4:**
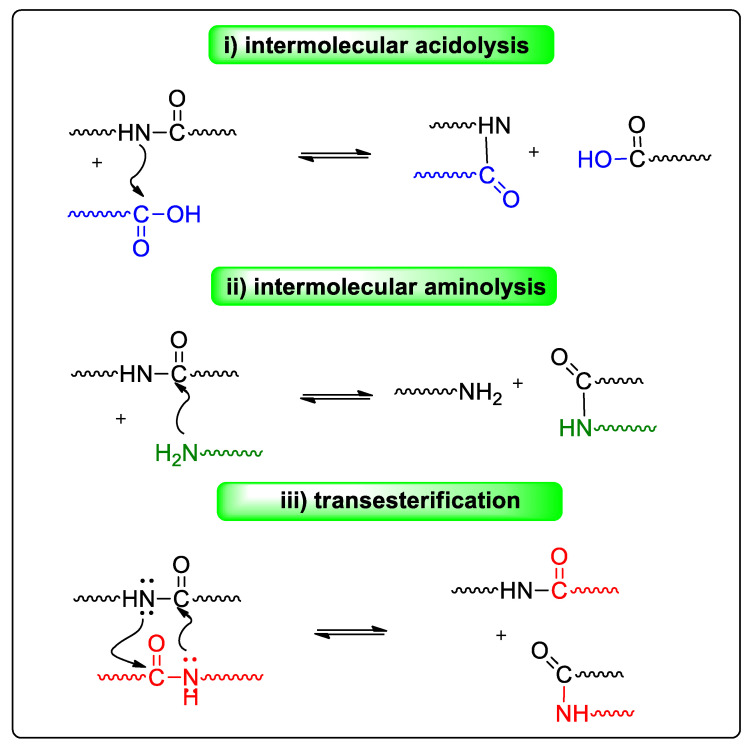
Mechanisms of the exchange reaction that takes place in polyamides.

The mechanism of CANs in polyamide is shown in Scheme 4 [57,58,59]. Three possible exchange reactions in which the chains are terminated by carboxyl or amine groups of the polyamides have been proposed. These reactions include: (i) intermolecular acidolysis, (ii) intermolecular aminolysis, and (iii) transesterification. Among these three routes, the most significant and efficient is intermolecular aminolysis. Like transesterification in polyesters, the presence of excess COOH functional groups is likely to promote the transamidation exchange.

**Transureafication:** Self-healing polyureas are widely investigated for applications in the coating industry. Transureafication facilitates the self-healing of polyureas. Excellent mechanical properties and self-healing capabilities that minimize costs are critically important for real-world applications [64]. A variety of reprocessable and self-healable polyurea-based materials with dynamic bonds can be prepared [65]. Various kinds of self-healable polyurea-based materials can easily be synthesized considering the availability of a wide range of isocyanates and amines feedstocks. These self-healing materials are expected to have considerable potential in a wide range of technical applications such as coatings (including smart coatings) and paints. The existing healable polyurea systems rely on hindered urea bond chemistry, which drives catalyst-free CANs in polyurea or urea-containing polymers [66].

**Scheme 5:**
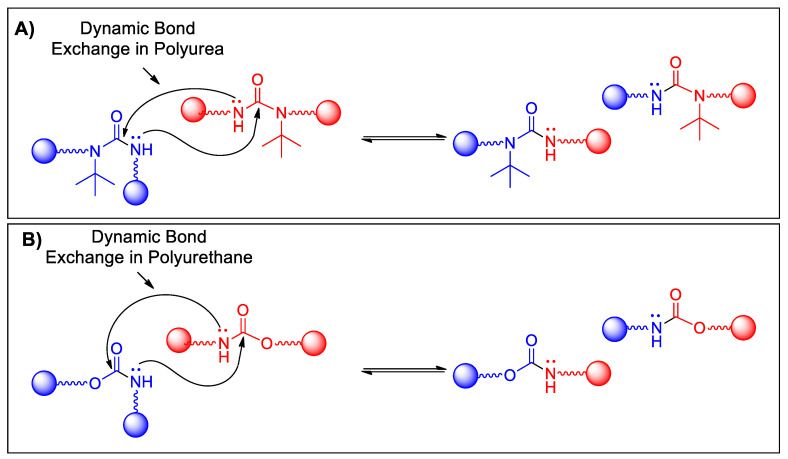
Dynamic bond exchange mechanism in: (**A**) polyurea and (**B**) polyurethane.

Scheme 5 represents the general mechanism of the dynamic bond exchange that takes place in urea systems [66,67,68]. Zinc catalysts increase the dissociation of urea via the formation of oxygen-bound zinc complexes. The resultant fast exchanging polyurea CANs can undergo rapid self-healing from the damage in the event of mechanical abrasion. In these cases, the self-healing could be readily controlled by adjusting the temperature. Various kinds of novel self-healing polyurea-based polymeric materials can be synthesized by this approach [69,70,71]. As a result, CAN polyurea-based materials are envisioned to have substantial applications in smart surfaces, smart electronics, and 3D printing [72].

**Figure 4:**
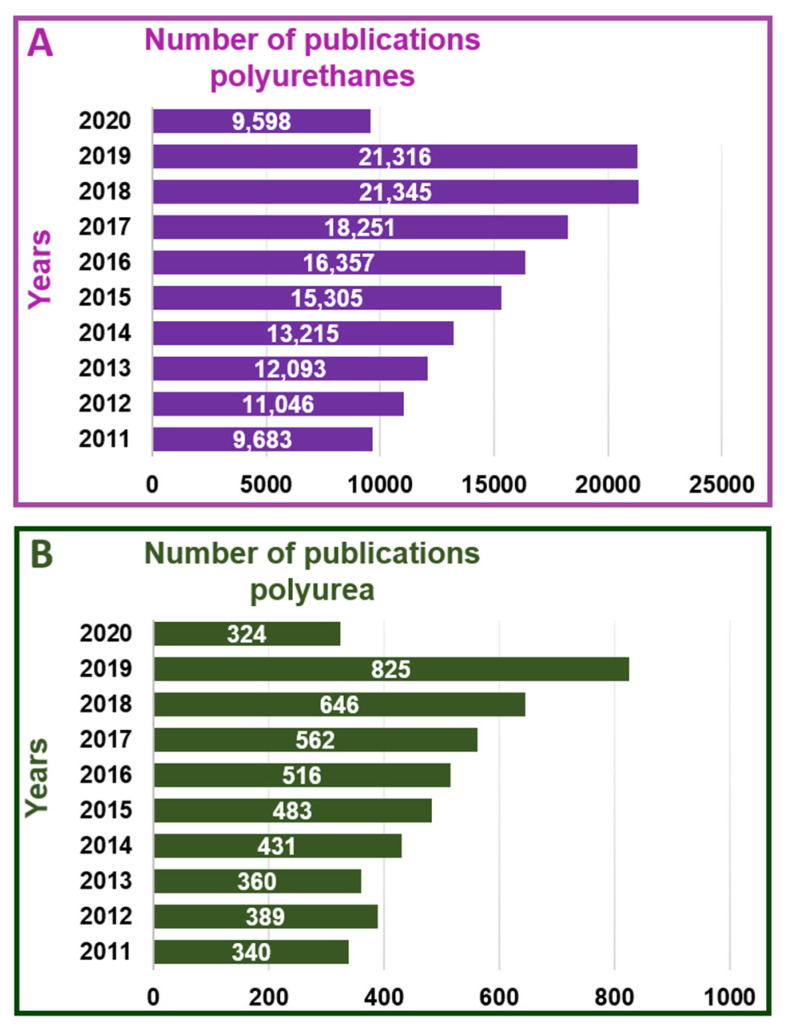
Articles published in the last 10 years related to: (**A**) polyurethanes; and (**B**) polyurea. These numbers were generated from the “Web of Science database” using the keywords “polyurethanes” for (**A**), and “polyurea” for (**B**). (Date data acquired: 15 July 2020).

**Figure 5:**
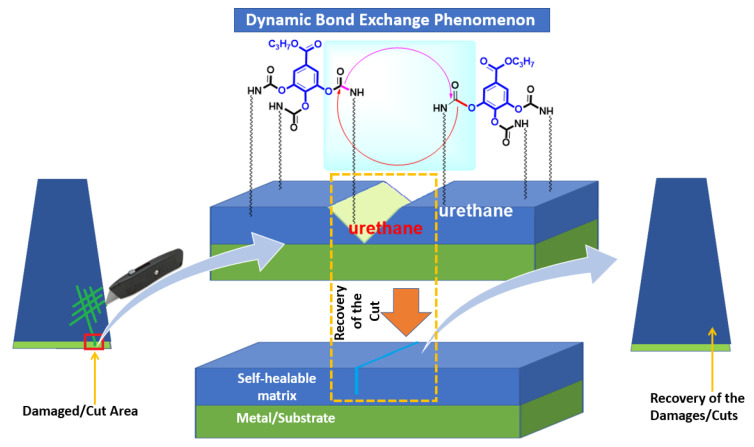
Dynamic bond exchange in self-healing polyurethane network [64].

**Transcarbamation:** Polyurethanes belong to the most versatile class of materials as they have numerous technical applications, and their properties can be readily tailored. In 2019 alone, more than ~21,000 articles were published on polyurethanes (Figure 4A). By varying the hard and soft domains with the use of a number of chain extenders, the mechanical strength and self-healing performance can be enhanced by inducing the formation of dynamic covalent bonds. Tailoring the nature of the hard phase is vital in order to synergistically enhance mechanical properties and self-healing efficiency. Coordinative bonds are helpful, as dynamic crosslinking joints can not only control chain displacement, but they can also provide better self-healing capabilities than hydrogen bonds. For example, the authors recently reported self-healing polyurethanes based on the transcarbamation chemistry of phenolic urethane bonds (see Figure 5). Reversible disulfide bonds also play vital roles in self repairable or self-healable materials, and they provide efficient platforms for the development of smart materials such as wearable devices, smart coatings, and flexible electronics [73].

## 2. Future Outlook and Conclusions

CANs offer a unique platform for sustainable functional materials with exciting applications in the field of self-healing as well as recyclable materials. For example, CANs provide a multitude of benefits, including: (i) increasing the longevity of materials; (ii) reducing maintenance costs due to their self-healing in nature; (iii) preventing critical failures; and (iv) reducing the amount of materials sent to landfills by enabling remolding and reuse of materials. Significant progress has been made with CANs and self-healing materials, but many challenges exist that should be addressed in order to enhance their applicability. For example, increasing the healing efficiency, achieving high healing rates, short healing/relaxing time, improving the healability at ambient temperatures, and improvement in the mechanical properties are the challenges that need to be addressed. If these drawbacks are successfully navigated, CANs will have numerous applications in a variety of demanding scenarios as high-performance materials for aerospace, automotive, machinery, underwater equipment, and so forth. In addition, the cost is a crucial consideration for practical applications, and therefore, more research related to the implementation of CANs and reversible functional groups in commodity materials such as polyolefins, polystyrene, and so forth is recommended. Some studies related to the use of CANs in commodity materials have already been undertaken, but further investigation will be needed in this direction [74]. Furthermore, the use of cost-effective and high-performance CANs materials will offer sustainable and zero-waste alternatives to non-recyclable conventional thermosets (e.g., epoxy esters and urethanes) and thus demands in depth research in this direction.

In conclusion, a brief overview of CANs system in polycarbonates, polyesters, polyamides, polyurethanes, and polyurea is presented with emphasis on their growth trends, market analysis, mechanistic insights, and potential applications. An outlook is given, which highlights the challenges as well as opportunities in CANs research. If the identified challenges are addressed, CANs will bring a paradigm shift in various sectors of real-world applications such as self-healing materials and the recycling and reuse of the crosslinked materials with enormous economic and environmental benefits.
